# An overview on aesthetic dentistry

**DOI:** 10.6026/973206300222451

**Published:** 2026-04-30

**Authors:** Vaibhav Awinashe

**Affiliations:** 1 Department of Prosthetic Dental Sciences, College of Dentistry, Qassim University, Saudi Arabia

**Keywords:** Aesthetic dentistry, cosmetic dentistry, restorative dentistry

## Abstract

A key goal of dental care is to restore teeth and craft smiles that are natural and visually appealing. Therefore, it is of interest to report this article to review advancements in aesthetic dentistry. Moreover, machine learning and artificial intelligence are expected to play a major role in automating treatment planning, smile design and aesthetic assessment in future.

## Background:

In recent years, there has been a demand for dental treatments driven by aesthetic concerns. Advancements in innovative techniques have empowered clinicians to better fulfill patients' cosmetic expectations. The concept of "aesthetics" has held importance since ancient times. The English and German terms "aesthetics" originate from the words "asthetisch" and "esthetique," meaning "the science of sensory perception" [1]. According to Garber and Salama, achieving dental aesthetics and an ideal smile relies on the harmonious relationship between the teeth, gingiva and the lip structure. Throughout history, healthy teeth have symbolized youthfulness. Health, beauty, and strength, and at times carried additional cultural significance [2]. A major aim of functional or corrective dentistry is to recreate the natural appearance of teeth as authentically as possible. Cosmetic dentistry broadly includes a variety of procedures. It includes veneers for fractured teeth, crowns, fillings, root canal treatments, and even tooth extractions. For example, following a tooth extraction, a bridge or implant is often planned to restore the most natural and pleasing smile possible. With the increasing popularity of cosmetic procedures, the importance of aesthetics in daily life has grown substantially. Cosmetic or aesthetic dentistry covers all dental treatments intended to enhance appearance. Although it is not classified as a distinct specialty within dentistry, achieving aesthetic outcomes remains a core objective of many dental interventions, alongside biological and functional considerations. Attractive smiles are often associated with positive social perceptions such as popularity, friendliness, intelligence, and elevated social status [3]. Therefore, it is of interest to review a historical perspective on the evolution of aesthetic dentistry. It highlights recent advancements in dental research and clinical practices.

## Historical development of aesthetic dentistry:

The desire to improve facial and dental appearance has been a consistent part of human culture since ancient times. Early civilizations recognized the value of an attractive smile, associating it with beauty, health, and social status. However, dentistry as an organized branch of medicine did not emerge until the 18th century, largely due to the pioneering contributions of figures like Pierre Fauchard. Who often referred to as the "Father of Modern Dentistry". His ground breaking work laid the foundation for the specialized treatment of both functional and cosmetic oral issues. The real transformation in aesthetic dentistry, however, occurred during the 20th century. This era witnessed rapid advancements in materials, techniques, and technology, leading to the development of procedures specifically aimed at enhancing the appearance of teeth and smiles. Innovations such as porcelain veneers, tooth-colored restorations, bleaching techniques, and implantology redefined the possibilities of cosmetic dental treatment. The increasing public demand for beautiful, natural-looking smiles and innovation, firmly establishing aesthetic considerations as a fundamental part of modern dental care [4, 5].

## Early era:

Aesthetic dentistry has its roots in the earliest human efforts to replicate the beauty of natural teeth. From the beginning, the goal was to imitate nature as closely as possible, both in form and function. Ancient civilizations, including the Egyptians, Etruscans, and Romans, made early attempts at dental restoration, using materials such as ivory, bone, gold, and even seashells to replace missing teeth. These primitive solutions reflected a deep appreciation for dental aesthetics and the social value placed on a healthy, attractive smile. Over time, the desire to restore not only the functionality but also the natural appearance of teeth became a driving force behind advancements in dental techniques. It helps for the evolution of modern aesthetic dentistry [2]. To closely imitate nature, aesthetic dentistry has developed from its fundamental principles, which are rooted in a deep understanding of the natural positioning, arrangement, shape, color, and proportions of teeth. Many academics evaluated this idea and associated criteria throughout many decades. It has been established by paleoodontological studies that cosmetic dentistry predates recorded human history. Certain types of cosmetic dentistry date back thousands of years [2]. The Upper Palaeolithic, around 11,000 BCE, is when the earliest records of dental restorations date back. Bitumen, vegetable fibres, and most likely hair were used to extend and replace the exposed pulp chambers of two anterior teeth in dental specimens discovered in Tuscany, Italy. Our understanding of restorative dentistry in ancient Greece or Egypt is lacking [6]. 7500-9000 years ago is when the earliest known evidence of human tooth drilling was found. Nine individuals' eleven permanently drilled molar crowns were discovered in Pakistan's Neolithic settlement of Mehrgarh [7]. The holes range in diameter from 1.3 to 3.2 mm and in depth from 0.5 to 3.5 mm. investigating these discoveries has even made it possible to rebuild potentially archaic enamel drilling equipment. Between 500 and 900 A.D., the Mayans practiced seven forms of dental modification, which included creating surface grooves, carving occlusal notches, and embedding inlays made from jade, turquoise, or pyrite [8]. The maxillary central and lateral incisors as well as canines are the teeth that are most commonly mutilated, according to Mexican anthropologist Romero. There is proof that the Mayas were skilled in implanting fragments of shell in place of lost teeth [9]. Evidence of dental ornamentation in Southeast Asia is demonstrated by an upper incisor specimen discovered at Halin, Burma. Chinese texts suggest that the "gold teeth" from Halin may be from the 7th or later century A.D [10]. Tooth alteration dates from the American Southwest and dates back to 880-1170 A.D. Several studies have documented dental alteration in historical African populations [2]. A significant milestone in the history of dental restorations occurred in 1460 when Giovanni d'Arcoli, a professor at the University of Bologna, became the first to use gold foil for filling teeth [11]. The Etruscans are credited with creating the first dentures in 700 B.C. by using bone and ivory. The teeth of either deceased animals or humans were used to make the majority of these early dentures. In 200 A.D., the Etruscans employed gold for the first time in dental crowns and bridges. The Egyptians replaced their teeth by hammering seashells into their gums.

## The evolution of modern cosmetic dentistry:

In the 1950s, metal was first fused to traditional porcelain crowns to enhance their aesthetics and durability. Early in the 20th century, dental labs began using acrylics and polymers for dentures. Acrylic dentures became available in 1930. In 1950, Buonocore introduced the enamel acid etching process, leading to the development of dentin bonding agents. The porcelain-fused-to-metal crown (Weinstein) was introduced in 1960. In 1970, Bowen developed Bis-GMA composite resin for jacket crowns and aesthetic restorations. The 1980s saw the advent of chairside CAD/CAM (Duret), intraoral scanning, commercial and at-home is whitening, ceramic acid etching, and laminate veneers. By 2010, zirconia crowns gained significant popularity (Table 1 - see PDF) [4].

## Smile in aesthetic dentistry:

The smile is a central feature in aesthetic dentistry. The goal of aesthetic dental restorations is to replicate or even enhance the natural appearance of the teeth. Fundamental aesthetic principles, such as proper alignment, symmetry, and proportion, form the basis of facial beauty. One key concept in smile design is the "golden proportion." This mathematical principle states that the ratio of the longer length to the entire length is equal to the ratio of the shorter length to the longer length [3].

## Aesthetic guidelines:

Aesthetic dentistry is built upon a deep understanding of the natural arrangement, proportions, positioning, shape, morphology, and color of teeth, aiming for the closest possible replication. Over the years, numerous authors have examined and articulated this concept, often drawing on their own subjective observations and findings [12]. Throughout the second part of the 20th century, a more concentrated effort was made to establish aesthetic standards for complete denture construction [13]. The first factor typically considered in a dental aesthetic assessment is the alignment between the dental midline and the facial midline. Tjan *et al.* (1984) classified smiles based on the extent of tooth visibility. A "low" smile or lip line is characterized by less visible tooth structure, while a "high" smile line refers to a greater amount of gingival exposure [14]. According to Williams (1914), there are three main shapes that human teeth can take: ovoid, triangular, and rectangular. He proposed that the facial outline should dictate the design of the teeth. Men should have square, angular teeth, and women should have ovoid, round, soft, and delicate teeth [15]. Many aesthetically disadvantaged patients need orthodontic treatment to achieve their goals regarding tooth position and angulation.

## Tooth color:

Both innate and exogenous colourations affect tooth colour. Extrinsic colour is defined by materials absorbed onto the surface of the tooth, whereas intrinsic colour is connected to light scattering and dentin and enamel absorption. Teeth colour is significantly impacted by demineralisation and dehydration. The viewer, the thing being observed, and the light source all affect how colour is perceived. In addition to hue, value, and chroma, the appearance of a tooth is influenced by secondary optical properties, including fluorescence, phosphorescence, opacity, translucency, iridescence, and surface gloss. Consequently, color-measuring tools and techniques have gained popularity, particularly in dental research to match the colour of restorations to the colour of surrounding teeth [3].

## Tooth whitening and bleaching agents:

Bleaching and whitening agents serve different purposes. Whitening addresses intrinsic pigmentation, while bleaching removes extrinsic stains. Bleaching works by generating free radicals, whereas whitening typically involves abrasive treatments. A prominent Persian polymath who lived between 854 and 925/932 AD. Rhazes, also known as Al-Razi, made significant contributions to various fields, including medicine, chemistry and philosophy. He is often regarded as one of the pioneers in the development of medical practices in the Islamic Golden Age. In relation to tooth whitening; Rhazes is credited with early formulations and methods aimed at improving dental appearance. He wrote extensively on the importance of oral hygiene, using a mixture of ingredients to clean and whiten teeth. Though the specific techniques used by Rhazes aren't fully detailed in historical texts, his work laid the foundation for many dental practices, including the idea of tooth whitening. For teeth whitening, Craton von Kraftheim (1519-1586) suggested oleumvitrioli, often known as ethyl ether [11, 16 and 17]. The earliest documented use of sodium chloride combined with chlorinated lime (calcium hypochlorite) as a bleaching agent date back to a meeting of the American Society of Dental Surgeons in 1848. In 1884, a bleaching solution was introduced, which required several minutes of contact with concentrated hydrogen peroxide (H2O2) to be effective. Further advancements were made by replacing water in the solution with ether, resulting in the creation of 25% hydrogen peroxide (Pyrozone) and 30% hydrogen peroxide (Superoxone) formulations [11].

## Adhesive restorations:

Adhesively bonded direct and indirect dental materials can correct tooth malformations, repositioning, and minor damage to restore aesthetics and produce a smile that is attractive. Dentin bonding agents, an acid etching method using 37% phosphoric acid, and resin restorations were introduced, leading to the development of adhesive restoration (Buonocore 1970). From the first generation to the current seventh generation, bonding agents were created later. Bonding agents have been developed to shorten processing times, eliminate smear layers, use fewer steps (multi-bottle procedures instead of single steps), and strengthen bonds. Fourth-generation bonding technologies included total-etch techniques that removed the smear layer completely (Kanca 1991). In fifth-generation adhesive systems, also known as 1-bottle systems, the primer-adhesive-resin solution is applied following a separate etch-and-rinse step. Self-etch adhesives were developed to eliminate the need for an additional acid-etch phase [3]. In 1963, Bowen introduced a groundbreaking tooth-colored resin known as BIS-GMA resin composite (bisphenol A, glycidyl methacrylate), which could be filled with various ceramic particles and quickly polymerized under oral conditions. Over time, many innovative and improved composite resin materials were developed, offering a range of compositions, colors, translucencies, and viscosities. Adhesive techniques and composite resin luting agents are now commonly used for bonded ceramic restorations, such as laminate veneers and inlays/onlays [3]. Composites using nanofilled resins have improved from self-curing to light-curing. Numerous tooth-colored restorative materials, including cention-N, ormocer, compomer, and zircomer, have been created with increased strength and aesthetics [18].

## Veneers:

Hollywood stars' looks were enhanced in the 1930s by Californian dentist Charles Pincus, who applied thin porcelain veneers to their front teeth [11].

## Prosthodontics:

Both fixed and removable prostheses have historically been made of a wide range of materials, with ceramics offering a desirable balance between durability and aesthetics. After the development of ceramics in the early 1960s, porcelain-fused-to-metal restorations rapidly became the standard for fixed single and multi-unit prosthetic restorations. Densely sintered, glass-infiltrated aluminium oxide ceramics were considered the first "high-strength" ceramic materials to demonstrate good clinical performance. The range of non-invasive and aesthetically pleasing treatment choices made possible by modern all-ceramic materials is extensive. When surgical procedures are not feasible, prosthetic management of cosmetic soft and hard tissue abnormalities using porcelain or pink acrylic may become necessary [3]. In comparison to PMMA polymer, methyl methacrylate monomer CDs, 3D printed entire denture CDs exhibited superior colour stability. Furthermore observed were the lifelike translucency, robustness, and great abrasion resistance of hybrid denture teeth packed with nanoparticles [18].

## Dental implants:

The discovery of osseointegration and the development of endosseous dental implants in the 1960s (Brånemark 1983) transformed the field of prosthodontics (Table 2 - see PDF) by providing a stable foundation for crowns, offering both anchorage and retention (Table 2 - see PDF) [19].

## Gingival and Periodontal esthetic approaches:

The position of the tooth in the arch, papilla height, gingival width, gingival biotype, and crown length are some of the variables that affect gingival aesthetics. The subject of periodontal plastic surgery has seen significant growth in interest in recent decades as a means of enhancing aesthetics. Gingival depigmentation, gummy grin repair, frenectomy, and papilla rebuilding are among the cosmetic changes. Recently, plasma therapy has been proposed as a novel treatment for gummy smile repair, gingival hyperpigmentation, and root covering treatments [1]. Recently, the technique of deep margin elevation (DME) has been developed as part of a treatment strategy that involves repositioning the cervical margin of teeth with subgingival defects to a supragingival location. This is achieved with a direct restoration to facilitate proper rubber dam isolation [20]. Connecting clinical tips related to deep margin elevation are listed in Table 3 (see PDF).

## Injection botulin toxin:

Botulinum toxin was used to treat an overactive muscle that was causing a gummy smile. It is easy to use, dependable, and produces instantaneous results. An alternative, more conservative method involves injecting Botox between the upper lip and nose to prevent the gums from showing when you smile. This works by paralyzing the muscles that cause the upper lip to lift when you smile. The benefit of this technique is that it does not require any surgical intervention [1].

## CAD/CAM technologies:

The advancement of computer-assisted technologies in diagnostics, treatment planning, design, and restoration fabrication has significantly impacted aesthetic dentistry by digitizing and simplifying key clinical and laboratory procedures [21]. Modern CAD/CAM systems are now capable of producing restorations ranging from single units to full-arch fixed and removable prostheses, in contrast to earlier systems, which were limited to inlays, onlays and single units [3].

## Digital smile design:

Cone beam CT and intraoral and extraoral optical scanners allow for a complete three-dimensional evaluation of all oral tissues and structures. Specialized software and computer programs facilitate digital planning and visualization of the desired aesthetic outcomes [3, 22 and 23].

## Present and future of aesthetic dentistry:

Libraries of natural tooth-and-smile algorithms now enable more visually pleasing results compared to traditional wax-ups or artificially created forms. The predictability and aesthetic outcomes are enhanced when teeth and smiles are designed through dynamic studies of facial features and lip movements. In a fully digital workflow, three-dimensional facial scans are integrated with intraoral, model, and cone beam CT images. Additionally, digital articulators and jaw-tracking sensors are incorporated into the latest smile design software.3. Looking ahead, artificial intelligence and machine learning are expected to automate much of the aesthetic evaluation, planning, design, and treatment processes. In the future, treatment planning, smile design, and aesthetic assessments are likely to be driven by AI and machine learning technologies.

## Conclusion:

Aesthetic dentistry has become a pivotal domain within clinical specialties, undergoing substantial advancements, particularly through the integration of digital technologies and workflows. These innovations facilitate a tailored, three-dimensional, interdisciplinary methodology for designing smiles.

## Advancement to knowledge:

The goal of aesthetic dentistry is to create harmonious smiles that resemble natural tooth aesthetics while maintaining dental health. It has developed from merely restorative dentistry to a sophisticated fusion of art, technology, and science. By 2026, the discipline will be propelled by developments in digital workflows (CAD/CAM and 3D printing), minimally invasive materials, artificial intelligence (AI) and the incorporation of facial aesthetics.

## Source of funding:

Self
